# Prolonged stable hypothermia during a 10‐hour cold open‐water marathon swim

**DOI:** 10.1113/EP093270

**Published:** 2026-01-21

**Authors:** Brendon H. Roxburgh, David T. Edgar, James D. Cotter

**Affiliations:** ^1^ School of Physical Education, Sport and Exercise Sciences University of Otago Dunedin New Zealand; ^2^ HeartOtago University of Otago Dunedin New Zealand; ^3^ Human Performance Lab New Zealand Defence Force Wellington New Zealand

**Keywords:** cold adaptation, cold‐water immersion, core temperature, core temperature monitoring, hypothermia, open‐water swimming

## Abstract

Marathon open‐water swimming presents extreme thermophysiological challenges, particularly in cold environments. This case report describes continuous core temperature data from a solo crossing of New Zealand's Foveaux Strait, an infamously cold (13°C –14°C), turbulent open‐water swim. A 52‐year‐old male swimmer (body mass index, 27.9 kg m^−2^; body fat, 18%) completed the 37 km swim in 9 h 52 min under standard marathon swimming rules (no wetsuit). Core temperature (measured via an ingestible thermometer pill) decreased rapidly after immersion, falling from 37.9°C to <35.0°C within 50 min. The swimmer reached a nadir of 33.88°C at 2 h 42 min and remained hypothermic for more than half of the swim (total, 369 min). Despite this, he displayed no overt cognitive or motor impairment, completed the swim unaided and did not experience an after‐drop post‐immersion. This case highlights the remarkable thermoregulatory tolerance of a cold‐adapted endurance swimmer.

## Abstract (highlights)

- **What is the main observation in this case?**
  A cold‐adapted marathon swimmer tolerated prolonged moderate hypothermia (core temperature <35°C for >6 h; nadir 33.88°C) during an open‐water swim in 13–14°C water.
- **What insights does it reveal?**
  This case demonstrates that in a trained and cold‐adapted marathon swimmer, prolonged immersion in cold water can be tolerated at core temperatures traditionally classified as clinically hypothermic, without functional compromise or adverse outcomes.

## 1 INTRODUCTION

Open‐water marathon swimming has grown in popularity, with an increasing number of amateur and elite athletes taking on extreme aquatic challenges. In Aotearoa New Zealand, one of the most formidable of these is the Foveaux Strait crossing, a 28–30 km stretch of cold, turbulent ocean separating Stewart Island/Rakiura from the South Island. The Foveaux Strait is regarded as one of the most technically and environmentally demanding swims, owing to its strong tidal flows, unpredictable weather, low water temperatures and regular presence of large marine life.

Swimmers attempting this crossing face substantial physiological and environmental risks associated with prolonged cold‐water exposure, most notably hypothermia (Saycell et al., 2019). Hypothermia, defined as a core temperature of <35°C, has been observed during open‐water swimming in water temperatures as high as 22°C (Brannigan et al., 2009; Castellani et al., 2006). In summer, Foveaux Strait water temperatures typically range between 11°C and 15°C, placing swimmers at high risk of heat loss. This risk is compounded by the strict equipment regulations for ratification by the New Zealand Open Water Swimming Association. In accordance with Marathon Swimming Federation rules (Federation, 2014), swimmers must complete the crossing without thermal protection (i.e., no wetsuit, only a textile swimsuit of standard coverage).

The ability to maintain core temperature throughout an open‐water swim in cold water is influenced by multiple factors, including body size and composition (particularly adiposity) (Drigny et al., 2021; Lounsbury & Ducharme, 2008), energy expenditure and swim pace (Drigny et al., 2021), environmental exposure and acclimatization to cold (Baldassarre et al., 2017). Greater subcutaneous body fat has consistently been shown to provide insulation, reducing the rate of heat loss during prolonged immersion and improving tolerance to cold water (Drigny et al., 2021; Pugh & Edholm, 1955; Saycell et al., 2019). In contrast, leaner swimmers might be at increased risk of hypothermia, particularly in swims taking several hours (Drigny et al., 2021).

This case report presents continuous core temperature measurements from a 52‐year‐old marathon swimmer throughout a Foveaux Strait crossing. Our aim was to document core temperature responses to this extreme cold‐water exposure. To our knowledge, this is the first published core temperature dataset from a Foveaux Strait swim, providing new insight into the thermophysiological demands of one of New Zealand's most iconic open‐water challenges.

## 2 MATERIALS AND METHODS

Core temperature data were collected in real time to support participant safety monitoring during the swim. Given the novelty of findings, the data were subsequently analysed retrospectively for inclusion in this case report. Written informed consent was obtained from the participant for both the use of these data and publication of the report.

### 2.1 Participant

The participant in this case study was a 52‐year‐old male. He has a 6‐year background in open‐water swimming and between 2019 and 2025, has completed 10 open‐water marathon swims ranging between 10 and 44 km in distance. The participant undertook a dedicated 5 month training and acclimatization period in preparation for the event, swimming three to five times per week (20−25 h weekly) in a public swimming pool and once per week (4–8 h) in open water. Additionally, the participant performed cold‐water plunges (as low as 10°C) daily (20−40 min per exposure). The participant's physical characteristics were as follows: body mass, 95.5 kg; height, 1.85 m; body mass index, 27.9 kg m^−2^; and body fat percentage, 18% as estimated by skinfold thickness (Ball et al., 2004).

### 2.2 Measures

Core temperature was measured continuously via an ingestible thermometer pill (e‐Celsius; BodyCap). The pill was ingested 3 h prior to commencing the swim. Core temperature was recorded every 60 s, with data stored internally within the ingestible sensor and later downloaded to an external receiver (e‐Viewer; BodyCap) following the swim. Total swim distance was measured using a global positioning system (Garmin Navionics) and water temperature measured (every 30 min) using an inbuilt thermometer on the escort boat.

### 2.3 Procedure

The Foveaux Strait crossing is 28 km from Saddle Point, Stewart Island/Rakiura to Stirling Point, Bluff. The participant completed the solo swim under the official rules of the New Zealand Open Water Swimming Association and the Marathon Swimming Federation. In accordance with these rules, he wore only a textile swimsuit, a single silicone swim cap and goggles. The rules prohibit the use of thermal or buoyant equipment (e.g., wetsuits) and forbid any form of artificial assistance, including physical contact with support vessels or drafting. The swimmer performed 2−3 min of dynamic flexibility exercise, targeting the shoulders and upper body, immediately prior to immersion. The swim commenced at 08.00 h in February 2025, with the participant supported by an escort boat. The swim was performed predominantly using front crawl with the head immersed in the water. The participant swam continuously for the first 60 min before taking an initial meal break, and thereafter paused briefly every 30 min, treading water for no longer than 30−40 s per break. During each break, basic cognitive function was assessed using a serial addition test. He also consumed water ad libitum and typically ingested ∼250 mL of a warm carbohydrate–electrolyte solution, hot chocolate or sweet tea fluid during the break. On alternating rest intervals, he also consumed carbohydrate–electrolyte gels, custard or marshmallows.

Immediately following water immersion on completing the swim, the participant was assisted onto a smaller support vessel and provided with a thermal blanket and a warm drink. Transport time from the immersion site to the primary escort vessel was ∼2–3 min. Upon arrival, the participant was dried with towels and provided with track pants, thermal undergarments and an insulated jacket. The return to harbour took ∼30 min, during which the participant remained seated and sheltered on the escort vessel.

## 3 RESULTS

The participant completed the Foveaux Strait swim in 9 h, 52 min and 9 s, starting at 08.00 h and reaching the mainland at 17.52 h. The total distance swum was 37 km at an average swim velocity of 1.07 m s^−1^. The temperature of water ranged between 13°C and 14°C. Air temperature was 11°C −18°C under cloudy skies, with wind speeds between 10 and 19 km h^−1^. The swimmer completed the swim without experiencing any adversity requiring medical attention.

**FIGURE 1:**
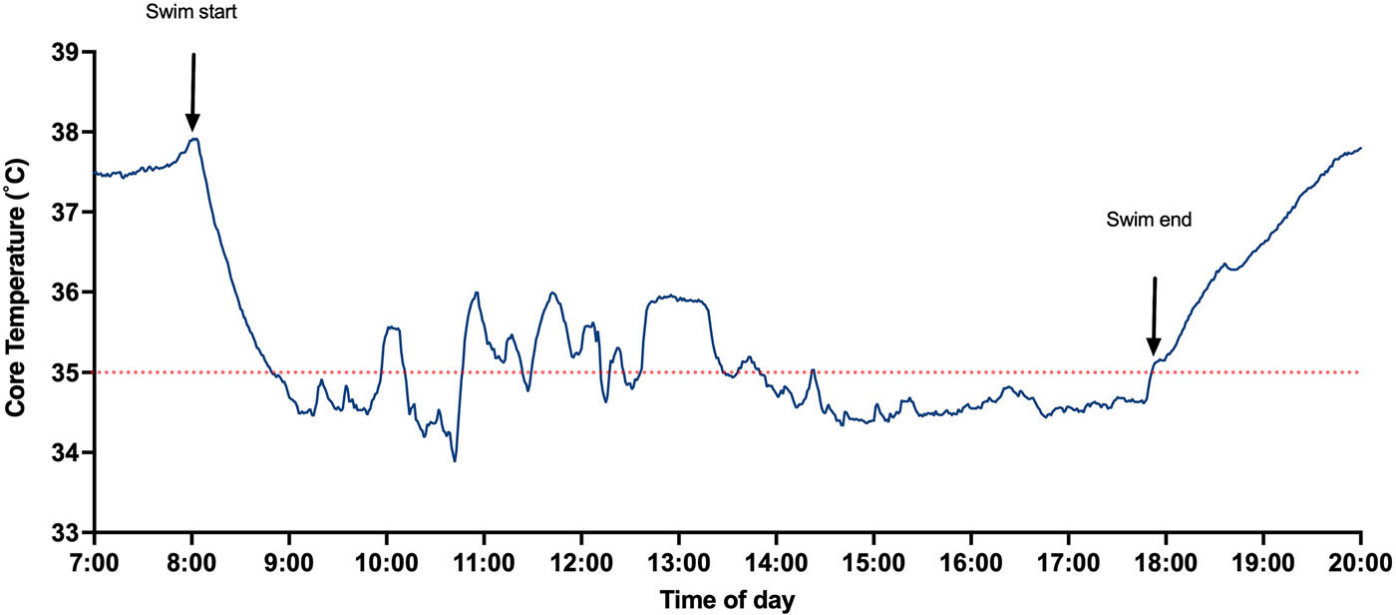
Core temperature before, during and after the swim. The *x*‐axis represents time (in hours), and the *y*‐axis shows core temperature (in degrees Celsius). A dashed red line indicates the clinical hypothermia threshold of 35°C.

Core temperature on entry to the water was 37.89°C (Figure 1). This temperature was maintained for ∼3−4 min before it decreased steeply. The participant was clinically hypothermic (core temperature of <35.0°C) within 50 min of swimming, reaching a nadir of 34.60°C (−3.42°C from baseline) at 65 min. The lowest recorded core temperature was 33.88°C after 2 h 42 min of swimming, that is, after one‐quarter of the duration. A total of 369 min (i.e., 62% of the swim) was spent with a core temperature of <35.0°C. The swimmer did not knowingly experience shivering during the swim, only shortly after extraction from the water.

## 4 DISCUSSION

This case study presents the first reported core temperature profile of a successful solo crossing of the Foveaux Strait, an open water marathon swim in cold conditions (13°C –14°C) and lasting nearly 10 h. The participant completed the swim in 9 h, 52 min and 9 s, spending >6 h and >60% of it in a clinically hypothermic state (core temperature of <35.0°C). Notably, core temperature fell rapidly during the first hour, eventually reaching a minimum of 33.88°C, yet stabilized and remained relatively steady for the remainder of the swim. This prolonged but stable hypothermic state challenges existing understanding of thermoregulatory limits during ultra‐endurance cold‐water exposure.

A steep early decline in core temperature, with clinical hypothermia within 50 min, is consistent with previous cold‐water swimming studies (Hayward et al., 1975; Keatinge et al., 2001; Noakes et al., 2009; Pugh & Edholm, 1955). Although metabolic heat production during swimming in 10°C water is ∼2.5 times greater than standing in the same water, the rate of core temperature cooling is ∼35% greater while swimming (Hayward et al., 1975). This is attributable to the movement of water across the skin increasing conductive–convective heat loss, especially for leaner swimmers and when wearing minimal thermal protection (Keatinge et al., 2001; Saycell et al., 2019). One case report of a swimmer (body fat, 35%; body mass index, 35.7 kg m^−2^) in 10°C water highlights the influence of body composition (Rüst et al., 2012). Rüst et al. (2012) reported an initial increase in core temperature (from 37.8°C to 38.1°C) across the first 20 min of swimming, before slowly and steadily decreasing to 36.3°C across 6 h. In the present case, core temperature decreased rapidly by 3.4°C within 65 min, before stabilizing at 34.6°C. The magnitude and speed of this decline is noteworthy and highlights the challenge of maintaining thermal balance during open‐water marathon swimming in cold water.

Remarkably, the participant remained in a clinically hypothermic state for >6 h of the swim (Castellani et al., 2006), apparently without functional or cognitive impairment and with no reported adverse effects or medical complications. Although previous studies have reported transient episodes of hypothermia in open‐water swimming (Keatinge et al., 2001), the sustained nature observed here is rare. Fatal hypothermia is likely after 3–9 h of immersion in 15°C water or after as little as 2–4 h in 10°C water (Tipton et al., 2022). This prolonged duration of hypothermia is unusual and highlights the exceptional physiological tolerance and possible adaptive mechanisms of experienced cold‐water swimmers.

The swimmer was likely to be well acclimatized, having regularly undertaken cold plunges lasting upto 40 min. Such habituation might blunt the typical sensory cues of thermal strain, increasing the risk of underestimating the onset or severity of hypothermia. Historical observations, including those by Pugh and Edholm (1955), highlight that subjective awareness of cold stress can be unreliable in well‐acclimatized individuals. In this context, non‐shivering thermogenesis might play a key role in sustaining core temperature, particularly in swimmers with a favourable body mass‐to‐surface area ratio, which reduces the rate of heat loss. Because non‐shivering thermogenesis draws on energy pathways separate from those required for swimming (van Marken Lichtenbelt & Schrauwen, 2011) and more centrally, it might help to maintain thermal balance without compromising mechanical output. Nevertheless, habituation and non‐shivering thermogenesis alone might provide a false sense of thermal status, reinforcing the need for objective temperature monitoring during prolonged cold‐water swims.

Several distinct perturbations in core temperature were observed throughout the swim. These changes were likely to be physiological in origin, rather than artefactual, given their duration and consistency with changes in environmental conditions. Notably, core temperature elevations appeared to coincide with sections of the swim marked by increased swell and current, suggesting a heightened metabolic response. The resulting increase in muscular activity and heat production might have contributed to transient rises in core temperature, despite the persistent cold‐water exposure (Drigny et al., 2021). These perturbations did not coincide with feeding times, and the temperature pill was ingested >5 h prior to the first large spike. However, we acknowledge that the possibility of measurement artefact (e.g., owing to unintentional fluid ingestion) in the initial period cannot be excluded. The second half of the swim was considerably calmer (based on swimmer feedback and direct observation), during which core temperature remained relatively stable.

Curiously, there was no observable after‐drop in core temperature after the swim, contrary to what has been reported in other studies involving cold‐water swimming (Noakes et al., 2009; Nuckton et al., 2000). This absence of after‐drop might be attributed to: (1) the prolonged and continuous nature of marathon swimming, which is likely to result in sustained peripheral perfusion throughout the body; (2) the low and stable core temperature preceding the egress; and (3) the lack of movement or passive heating of skin following egress. Unlike sudden or brief cold exposures, where vasoconstriction protects core temperature and then rewarming causes a delayed drop, the extended duration of cold exposure in marathon swimming might prevent such central–peripheral thermal gradients from developing. As a result, tissue temperatures might equilibrate more gradually during the swim itself, reducing the thermal shock and redistributive cooling that typically underlies the after‐drop phenomenon.

## 5 CONCLUSION

This case demonstrates that in a trained and cold‐adapted marathon swimmer, prolonged immersion in cold water can be tolerated at core temperatures traditionally classified as clinically hypothermic, without functional compromise or adverse outcomes. The sustained hypothermia, rapid early cooling and absence of after‐drop observed here challenge conventional assumptions about thermoregulatory response and limits.

## AUTHOR CONTRIBUTIONS

Study conception: Brendon H. Roxburgh, David T. Edgar and James D. Cotter. Data collection: Brendon H. Roxburgh and David T. Edgar. Data interpretation: Brendon H. Roxburgh, David T. Edgar and James D. Cotter. Manuscript writing: Brendon H. Roxburgh and David T. Edgar. All authors have read and approved the final version of this manuscript and agree to be accountable for all aspects of the work in ensuring that questions related to the accuracy or integrity of any part of the work are appropriately investigated and resolved. All persons designated as authors qualify for authorship, and all those who qualify for authorship are listed.

## CONFLICT OF INTEREST

None declared.

## FUNDING INFORMATION

None.
